# In Quest for Improved Drugs against Diabetes: The Added Value of X-ray Powder Diffraction Methods

**DOI:** 10.3390/biom7030063

**Published:** 2017-08-22

**Authors:** Fotini Karavassili, Alexandros Valmas, Stavroula Fili, Christos D. Georgiou, Irene Margiolaki

**Affiliations:** Section of Genetics, Cell Biology and Development, Department of Biology, University of Patras, GR-26500 Patras, Greece; fkar@upatras.gr (F.K); valmas@upatras.gr (A.V); filistavr@gmail.com (S.F); c.georgiou@upatras.gr (C.D.G)

**Keywords:** insulin, phenolic derivatives, crystallography, powder diffraction

## Abstract

Human insulin (HI) is a well-characterized natural hormone which regulates glycose levels into the blood-stream and is widely used for diabetes treatment. Numerous studies have manifested that despite significant efforts devoted to structural characterization of this molecule and its complexes with organic compounds (ligands), there is still a rich diagram of phase transitions and novel crystalline forms to be discovered. Towards the improvement of drug delivery, identification of new insulin polymorphs from polycrystalline samples, simulating the commercially available drugs, is feasible today via macromolecular X-ray powder diffraction (XRPD). This approach has been developed, and is considered as a respectable method, which can be employed in biosciences for various purposes, such as observing phase transitions and characterizing bulk pharmaceuticals. An overview of the structural studies on human insulin complexes performed over the past decade employing both synchrotron and laboratory sources for XRPD measurements, is reported herein. This review aims to assemble all of the recent advances in the diabetes treatment field in terms of drug formulation, verifying in parallel the efficiency and applicability of protein XRPD for quick and accurate preliminary structural characterization in the large scale.

## 1. Introduction

Diabetes mellitus (DM), was one of the first diseases ever described [1], whereas its name, was originated from the Greek word “diabaino” (=passing through referring to the great emptying of the urine) and “meli” (=honey referring to the sweet taste of the patients’ urine due to high glucose concentration). Patients suffering from this disease (type I or II) lack control of glucose metabolism, due to inadequate levels and/or function of human insulin (HI). Unfortunately, until Banting and Best’s isolation of insulin-containing extracts in 1926 [2], the prognosis for a patient was no better than it was over 3 millennials ago. Until now, significant effort has been dedicated on the production of therapeutics for the control of DM and its treatment [3,4], alleviating the daily routine for millions of patients. However, diabetes, affecting nowadays a large and steadily increasing part of the world population [5,6], causes increased morbidity and mortality, and its global impact is likely to accelerate over the coming decades.

While type I DM (“insulin-dependent diabetes mellitus” or “juvenile diabetes”), which results from the pancreas’s failure to produce enough insulin, reduces life expectancy of patients to almost 13 years, type II DM (related to failure of cells to respond properly to insulin) is not a directly life-threatening disease. Patients, however, experience a number of malfunctions of their circulatory system (hypertension, abnormal levels of cholesterol, triglycerides, and blood sugars) as well as obesity [7,8], conditions which frequently induce numerous implications in human’s health, such as nephropathy, neuropathy, and several cardiovascular diseases (CVDs), including coronary heart disease (CHD), and stroke and cardiomyopathy [9]. Recently, free radicals as well have been reported to intervene in the onset of diabetes [10], while radical scavengers (such as salicylates) appear to effectively prevent diabetes and reduce the severity of diabetic complications and especially myocardial infractions [11,12].

Although there have been many improvements in diabetes’ therapy, it still remains a demanding challenge. Today, fifty years after ΗΙ’s primary structural characterization [13], millions of patients rely on external provision of this essential pancreatic hormone of 5808 Da for their survival. Hypodermic injections, initially employed 80 years ago, containing microcrystals or balanced proportions of microcrystals and amorphous hormone, can achieve glycemic control over a certain period of time, depending on the timeframe of their action.

A rapid-acting insulin product begins to act within 15 min after injection, reaches maximal levels in about 1 h, and continues to act for 2 to 4 h (e.g., insulin glulisine-Apidra, by Sanofi-Aventis Deutschland GmbH, Frankfurt, Germany), while a short-acting one enters the bloodstream within 30 min after injection, and peaks from 2 to 3 h later (e.g., Novolin R, by Novo Nordisk A/S, Copenhagen, Denmark), and a long-acting one enters the bloodstream several hours after injection and exhibits a glucose-lowering profile over a 24-h period (e.g., insulin detemir-Levemir, by Novo Nordisk). Slow dialysis of the injected clusters causes dissociation of insulin’s hexameric structural motif (the typical storage form of crystalline insulin [14]) into dimers and monomers, capable of diffusing through interstitial fluid, penetrate the capillary wall, and enter the blood circulation.

The aforementioned insulin microcrystals contain a mixture of polymorphs, distinguished either by the number of zinc ions per hexamer (two or four), or by the three distinct B chain conformations (extended-intermediate-helical) of the insulin monomer, denoted as “T”, “R^f^”, and “R”, naming the hexamers as T_6_, T_3_R_3_^f^ and R_6_, respectively [15,16,17]. T_6_ hexamers typically occur in the presence of zinc [18], while, the addition of chloride and thiocynate anions results in T_3_R_3_^f^ hexamers [19,20]. Phenolic derivatives (phenol, resorcinol, *m*-cresol, etc.), acting as ligands, enter inside corresponding pockets and strongly stabilize the hexameric form by hydrogen bonding the carbon oxygen of Cys^A6^ with the amide proton of Cys^A11^ [21], while in parallel assist towards the acquisition of the R_6_ conformation, where residues B1–B8 of each molecule adopt an α-helical configuration [22]. However, this transition is not always feasible [23,24], while there is the unique, until now, case of the insulin analogue “ultralente” [14,25], where the addition of methylparaben, resulted in the reverse structural transition (T_3_R_3_^f^ ➔ T_6_) [26]. The structural behavior of HI in the presence of several organic additives, mainly phenolic derivatives, which were originally used in pharmaceutical formulations as preservatives due to their strong antimicrobial activity, has been extensively examined [20,27,28,29].

B-chain conformational changes arise as well with respect to pH. This phenomenon is closely related to the charge ionizable groups of insulin adopted in distinct pH values. At pH values higher than 7.5, all carboxyl groups are negatively charged, one arginine and one lysine positively charged, all four Tyr and two His amino acids are neutral, while the two amino termini are partially or largely unprotonated, depending on the exact pH [30].

Previous studies have established pH as an important factor strongly affecting protein crystallinity as well [31,32]. It is known that protein solubility reaches a minimum near the isoelectric point (pI), whereas solubility increases in both directions away from it. Within a pH range—particular for each protein—molecules are altered in various ways. At certain pH values either Lys or Arg side chains start losing their positive charge or, in an alternative case, carboxyl groups of Asp and Glu side chains lose their negative charge [33]. Such partial amino acid neutralization disrupts the formation of salt bridges between protein molecules, and thus decreases the crystallization rate. Lower nucleation levels usually lead to the formation of fewer but larger, and better-shaped crystals owing to the control of rapid crystal growth at low and high pH values.

Since the first crystallographic characterization of insulin, a number of polymorphs have been identified as often related to distinguishing states of insulin [15,19,34]. The continuous study of human insulin’s structural behavior (wild-type or various mutants) [20,35], and the enormous amount of information extracted, allows for the synchronization of crystalline insulin dissociation inside of the human body. Despite the resulting availability of various insulin products with rapid, intermediate, or prolonged action profiles, there is always a need for further improvement, as well as novel crystalline phases to be discovered [36]. Knowledge of the crystalline properties of individual polymorphs and their direct linkage with drug’s Absorption, Distribution, Metabolism, and Excretion (ADME) characteristics can lead to the production of more efficient formulations, or even lead to the development of insulin preparations with alternative ways of delivery, such as sustained-release formulations or inhaled compounds [37].

As insulin products ideally contain millions of microcrystals, these should be studied in unity, rather than by isolating individual crystals to perform experiments, as single crystal X-ray diffraction (SCXD) approaches require. In contrary, powder diffraction methods can offer a holistic exploration of the system under investigation, and can quickly provide accurate information while screening different polymorphs, advantages that clearly point out the value of this approach. Until the development of the Rietveld method [38], initially for neutron powder diffraction data and later for X-ray powder diffraction (XRPD) data [39], the most common practices of this method included phase identification and quantitative phase analysis, especially in industrial settings [40,41]. However, powder diffraction currently holds a wider spectrum of applications, including structure determination of zeolites, small organic molecules, and most recently, biological macromolecules [42,43,44,45,46,47].

The continuous evolution during the last fifteen years of macromolecular powder techniques has considerably improved the quality of data, which are routinely collected from microcrystalline precipitates using synchrotron or laboratory sources [48,49,50]. The simplicity of XRPD data collection, and the uniqueness of the pattern obtained from each polymorph suggest that the XRPD technique as the most suitable approach for carrying out a quick and reliable characterization of various microcrystalline precipitates [51,52]. This approach may provide medium resolution structural models (3–10 Å), however it offers the ability to study low quality crystals, polymorph screening becomes a routine practice, and time-resolved studies are also possible. In addition, the size of the individual crystallites composing the powder can be directly estimated from the peak widths present in XRPD profiles. Further advantageous aspects deriving from the use of powder diffraction include homogeneity and purity control [45,53] of the precipitates—valuable features for the pharmaceutical industry that cannot be obtained by SCXD measurements, as a single crystal may not be representative of the entire batch.

The main purpose of this review article is to summarize an extensive series of experiments and findings of the last decade, while investigating the structural behavior of human insulin. Results include the structural characterization of HI in the presence or absence of small organic molecules (ligands) in a wide pH range (~4.5–8.5) obtained via XRPD, including the identification of four previously unknown insulin polymorphs: *C*222_1_, *C*2, *P*2_1(α)_ and *P*2_1(γ)_ [29,53,54,55,56]. These studies indicate that there is still a rich diagram of human insulin phase transitions, as well as novel crystalline phases to be discovered.

## 2. Overview of Insulin Crystalline Structures

A broad selection of ligands was included in distinct crystallization experiments towards growing insulin crystal complexes, which were systematically studied under pH alterations. Polycrystalline precipitates (~50–100 μL volume each) of complexes, in the presence of zinc ions, were produced using the batch method, for almost all conditions studied.

Structural results, described in detail in the following sections, were obtained from diffraction patterns employing different instrumentation (Synchrotron sources: National Synchrotron Light Source (NSLS), MAX-lab synchrotron, European Synchrotron Radiation Facility (ESRF), Swiss Light Source (SLS) and laboratory diffractometers: RU200 (Rigaku Ltd., Tokyo, Japan), X’Pert PRO (PANalytical BV, Almelo, The Netherlands)) in order to optimize data quality with respect to angular resolution (FWHM) and d-spacing range.

Prior to powder diffraction data collection, polycrystalline samples were loaded into borosilicate glass capillaries, sealed with grease to prevent dehydration, mounted on the diffractometer, and spun to ensure adequate powder averaging. Synchrotron X-ray diffraction (XRD) experiments were carried out by applying capillary translation among scans in order to eliminate radiation damage effects (alterations in unit-cell parameters, peak broadening, etc.) owing to the intense synchrotron beam. Identical scans collected from the newly exposed parts of each sample were summed together based on long-established methods [57], in order to increase the counting statistics without degrading data quality. In cases where laboratory instrumentation was employed, the powder data didn’t exhibit any radiation damage, even after 24 h of constant irradiation. The extraction of reliable lattice values and characterization of the peak shape and background coefficients was achieved via the Pawley method [58].

### 2.1. First Human Insulin XRPD Studies

Following the first successful experiment with polycrystalline metmyoglobin, conducted by R.B Von Dreele [44], which demonstrated that protein structure refinements using XRPD data are feasible, his research was further extended to insulin. Initially, microcrystalline slurry was produced as a byproduct of a single-crystal sample [59] by grinding the crystals with mother liquor in an agate mortar [60]. The slurry was placed in a glass capillary, and XRPD data were collected while the capillary was spun. Data collection was performed at room temperature at X3b1 beamline, at the National Synchrotron Light Source, equipped with a double Si(111) monochromator and a Ge(111) analyser.

**Figure 1:**
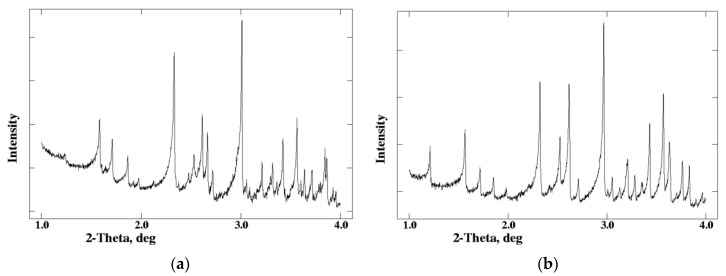
X-ray powder diffraction (XRPD) patterns of Zn-human insulin collected with λ = 0.700233 Å. (**a**) XRPD pattern of freshly ground Zn-human insulin complex (T_3_R_3_DC); (**b**) XRPD pattern of aged Zn-human insulin complex (T_3_R_3_^f^); the pattern shown was produced by the sum of two individual scans collected at 2 s·step^−1^ and 0.002° step^−1^ (Reproduction of Figure 1 from reference [60]. Reproduced with permission of the International Union of Crystallography).

From freshly made slurry, the diffraction pattern shown in Figure 1a was obtained; whereas, material left for 3 days after grinding produced a distinctly different diffraction pattern as shown in Figure 1b. The pattern from the ground material was indexed in rhombohedral symmetry, with *a* = 81.9678 (7) Å, *c* = 37.5914 (8) Å, identical to the single-crystal unit cell for T_3_R_3_^f^ HI conformation [19], whereas the pattern from the freshly ground material, revealed a previously unknown rhombohedral polymorph with *a* = 81.2780 (7) Å, *c* = 73.0389 (9) Å, which is fundamentally a doubled c axis superlattice of the T_3_R_3_^f^ structure (a phase denoted as T_3_R_3_^f^DC).

Owing to the close relationship between these two phases, the structure solution of T_3_R_3_^f^DC using the molecular-replacement technique was employed. A starting model was introduced from the single-crystal coordinates for the T_3_R_3_^f^ complex [19], and a three-parameter (two rotation angles and one translation) rigid-body Rietveld refinement was later performed. Atomic coordinates, extracted from stereochemically restrained Rietveld refinement of the T_3_R_3_^f^ crystal structure, were used to complete the rigid-body refinement of the T_3_R_3_^f^DC.

**Figure 2:**
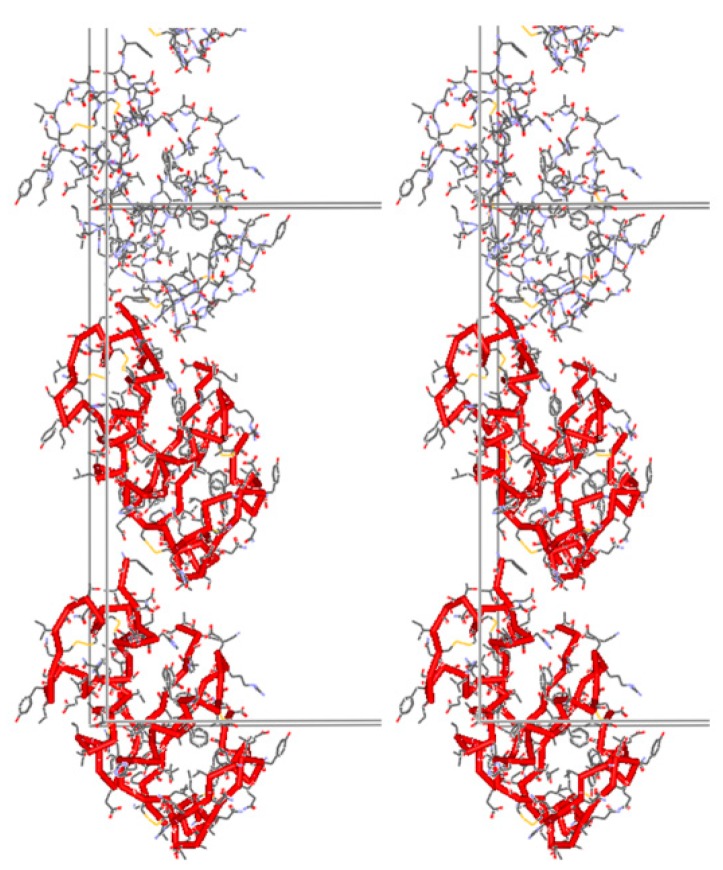
Packing of three insulin dimers arranged alongside c axis in T_3_R_3_^f^DC structure. A Ca trace is colored red and unit-cell boundaries are also visible (Reproduction of Figure 4 from reference [60]. Reproduced with permission of the International Union of Crystallography).

The complete structural characterization of the T_3_R_3_^f^DC insulin form achieved via XRPD was also verified via single crystal experiments one year later [59], and revealed a number of special features of this new variant of the T_3_R_3_^f^ human insulin-Zn complex. After grinding, a reduction of the material’s volume by 2.095% or 1490 Å^−3^ per T_3_R_3_^f^ complex was evident, which consequently induced a structural change resulting in c axis doubling of the rhombohedral unit cell. One of the independent dimers rotates 17.2° about the c axis in the conversion from T_3_R_3_^f^ to T_3_R_3_^f^DC; the other rotates 9.5° in the same direction (Figure 2). This rotation is probably associated with a collapse of the spacing between the pairs of (AB)_2_ complexes along the crystallographic c axis, and a repositioning of B chains with extended conformation. Conceivably, water molecules extracted from the structure during grinding could originate from this particular location.

This was one of the first research results demonstrating the applicability of powder diffraction method for macromolecular crystal screening and detailed structure solution of a protein molecule. Within the next five years, continuous developments in instrumentation as well as in data collection and analysis were carried out in parallel by Robert Von Dreele at Argonne National Laboratory (USA) and Irene Margiolaki and colleagues at ESRF (Grenoble, France). Their early studies on lysozyme (Turkey or Hen egg-white) as a model system further established the use of XRPD as a valuable tool in the identification of small structural variations in protein molecules [49,61,62,63].

### 2.2. Characterization of Distinct Insulin Formulations Via XRPD

Along with the underlying difficulties of developing and producing biopharmaceutical compounds, the characterization of the final product can sometimes be even more challenging and demand a repeated revision process of analytical methods performed in a high throughput manner, without compromising the accuracy of the obtained results. On top of this, protein therapeutics correspond to a class of products which have an intricate structure whose integrity determines the bioavailability, biological activity, clinical efficacy, and safety. All factors which control the aforementioned characteristics of a product are extensively studied in the production processes, and provide valuable information for further refining the enzyme/protein manufacturing.

The first study of this kind was originally conducted in 2006 by Norrman et al. [54], where 12 insulin formulations (some commercially available) were investigated via XRPD. Despite the medium-resolution XRPD patterns obtained, the data in combination with multivariate data analysis were used to compare insulin microcrystals preparations.

The commercially available insulin preparations examined in that project (Ultratard, Ultralente, Lente, Detemir, Penmix30, Novomix30 and Protaphan) were obtained from Novo Nordisk A/S, whereas additional microcrystals were prepared following the batch crystallization method. All products examined were “descendants” of the first stable protracted insulin formulation, the Neutral Protamine Hagedorn (NPH), which was introduced in 1946 [64]. This formulation was based in an observation by Hans Christian Hagedorn (founder of former Novo Nordisk A/S) and B. Norman Jensen in 1936, introducing that the effects of injected insulin could be prolonged with the addition of protamine—a peptide consisting mainly of arginine—obtained from the semen of river trout. An insulin–zinc solution was cocrystallized with protamine, reducing insulin’s solubility and resulting in NPH insulin; an intermediate–acting insulin product.

Among all HI crystals produced by batch crystallization, two novel crystal types were obtained. Orthorhombic *C*222_1_ crystals (*a* = 59 Å, *b* = 219 Å, *c* = 223 Å) with three hexamers in the asymmetric unit, adopting the R_6_ configuration were identified in presence of urea, NaCl, and resorcinol at pH 6.7 [54], whereas in slightly higher pH values (~7) monoclinic *C*2 crystals (*a* = 100 Å, *b* = 60 Å, *c* = 62 Å, *β* = 116°) were observed containing one hexamer with R_6_ molecular conformation in the asymmetric unit, and 50% solvent content [55]. Crystallization conditions for all formulations used in that study are summarized in Table 1.

Protein powder data of this study were collected at room temperature, both in-house (on a Mar345 imaging plate detector, using an RU200 rotating anode, λ = 1.5418 Å, Rigaku Ltd.) and at the MAX-lab synchrotron (Lund, Sweden), beamlines 711, 911-2 and 911-3 [66,67], using charge-coupled device (CCD) detectors. Data indexing was in all cases unsuccessful, even though a variety of software was exploited, due to low angular resolution (broad overlapping diffraction peaks) and the use of area detectors, which resulted in further peak overlap. Thus, only synchrotron powder diffraction patterns were employed for extracting preliminary structural information, due to their advantageous d-spacing and angular resolution. Nevertheless medium-resolution powder diffraction patterns were enough for effective classification in crystal systems via Principal Component Analysis (PCA) [68]. Crystallographic properties of all samples described in this project are listed in Table 2.

**Figure 3:**
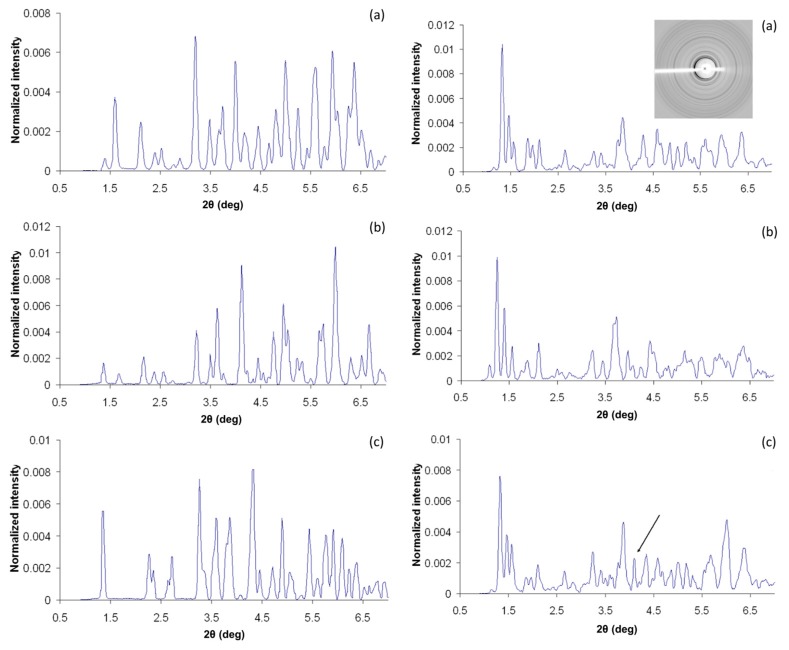
Comparison of experimental diffraction patterns of various crystal types. Left Panel: XRPD patterns (normalized intensities) of crystals D (**a**), E (**b**) and F (**c**), in *R*3 space group. Several variations in peak positions are evident in a wide 2θ range, owing to distinct B-chain conformations (R_6_, T_3_R_3_^f^ and T_6_). Right Panel: XRPD patterns obtained from I (**a**), J (**b**), and K (**c**) crystals in *P*4_3_2_1_2 space group. I and K patterns are similar, apart from an extra peak found in the K dataset (indicated with an arrow), while larger unit-cell parameters of J crystals are clearly depicted as numerous shifts in diffraction peaks positions (Reproduction of Figures 2 and 3 from reference [54]. Reproduced with permission of the International Union of Crystallography).

Patterns from different insulin polymorphs showed distinct peaks in the low 2*θ* region (0.9° to ~6°). Visual evaluation of the plots in Figure 3 shows that crystals, belonging to the same crystal system according to the bibliography with the same type of structure, have very similar powder patterns as well. Despite the fact that powder patterns have been collected without the optimum instrumentation, they reveal even small differences in protein structure based in alternations in peaks’ positions (F, D, and E crystals), and/or the extinction of several peaks (I, J, and K).

F, D and, E crystals all belong to the rhombohedral space group *R*3 with T_6_, R_6_ and T_3_R_3_^f^ molecular conformations, respectively. As seen from Figure 3 (left panel), similar peaks in the three patterns are generally shifted by less than 0.12° in 2θ. Peak variances are more evident within the 2θ range of 3.95 to 4.35°, where in all cases a high-intensity peak is observed, but its position is clearly different. The shifts in peak positions are associated to structural differences in the N-terminal part of the B-chain, causing alternation in crystal packing and thus in the unit cell constants; especially in the length of the c-axis.

Crystals I, J, and K belong to the same space group according to Table 2. Powder patterns from the three types of crystallites share a high degree of similarity, especially in the low 2θ region, as shown in Figure 3 (right panel). The major difference among them is an additional peak at 2θ = 4.1° in the K pattern (marked with an arrow in Figure 3c) that is not found in the I pattern. Also, peak positions in the J pattern are shifted relative to I and K patterns in the entire region, reflecting the slightly larger unit-cell parameters of J crystals (Table 1). This can be explained considering the mutation B28Asp in J crystals, which alters the molecule’s charge, thus a higher proportion of the protamine peptide is being bound on insulin [65], resulting in slightly altered unit-cell parameters.

Visual analysis of the powder patterns described above, demonstrates that even without successful data indexing, the method can be used to effectively distinguish different crystal systems and assess homogeneity of different batches or preparations of insulin. However, the complexity increases when examining a plethora of microcrystal suspensions, and the procedure can be time-consuming, thus Norrman et al. [54], employed the PCA analysis to facilitate the interpretation of powder patterns. Through PCA, data dimensionality (number of variables) is reduced, via a statistical procedure, from several hundreds to two or three principal components, resulting in a visual representation of the relationships and similarities of the—powder patterns of the—samples, by grouping them into clusters. Diffraction patterns from the crystals mentioned above were represented as data points, and their clustering indicated a high similarity feature within each group. For example, the relative shifts in peak positions of the three rhombohedral D, E, and F crystals, due to distinct B-chain conformations (R_6_, T_3_R_3_^f^ and T_6_ respectively) had a large impact on the distribution of their PCA scores in the plot, and thus were not clustered together. Following this approach, different crystal systems and/or structural arrangements can be clearly separated, further facilitating the detection of novel polymorphs as in the case of B and X type of crystals, which were clearly distinguished from other clusters.

**Figure 4:**
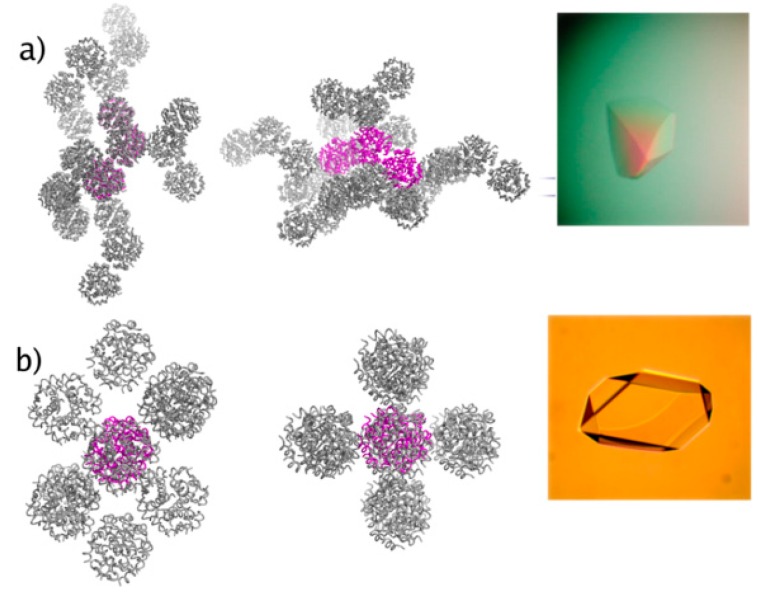
Structural models derived from single crystal methods. Packing of (**a**) *C*222_1_ and (**b**) *C*2 insulin hexamers. The asymmetric unit is denoted in both cases with magenta. Single crystals of the *C*222_1_ and *C*2 forms are shown in the right (Reproduction of Figure 1 from reference [55]).

The identification of two novel crystal forms (orthorhombic *C*222_1_ and monoclinic *C*2, Figure 4) of human insulin accomplished in this project declare the use of XRPD as a powerful approach for characterization and evaluation of macromolecular microcrystalline suspensions, both during polymorph screening, and in manufacturing process control. The medium-resolution data of the early XRPD era did not allow for detailed structural characterization, thus this was achieved a year later [55] via single crystal experiments (Protein Data Bank (PDB) codes: 2OM1 for the *C*222_1_ crystal form and 2OLZ for the *C*2 crystal form).

The discovery of novel insulin polymorphs from Norrman & Schluckebier [55] triggered the research around insulin, and variations in cocrystallization and pH conditions forced the discovery of several other insulin crystalline polymorphs waiting to be examined in terms of physical stability, dissolution rate, and other bioavailability properties.

Bovine insulin polycrystalline precipitates were extensively studied later on as well, in a wide pH range 5.0–7.6. Powder X-ray diffraction data revealed the T_6_ hexameric insulin form (space group *R*3; unit-cell parameters *a* = 82.5951 (9) Å, *c* = 33.6089 (3) Å for the sample crystallized at pH 5.0) in agreement with the high-resolution structure of HI, identified earlier by single crystal experiments [69,70]. 

Fourteen powder diffraction profiles with slightly different lattice parameters were selected for structure analysis. Lattice parameters variations were caused by alterations in the sample preparation procedure, or were induced by radiation exposure. In the diffraction patterns, these variations are depicted by shifts in the positions of adjacent peaks, allowing the contributing reflections of the overlapped peaks to be partially deconvoluted. Stereochemically restrained Rietveld refinement was performed to obtain an average crystal structure of bovine insulin over the pH range using the General Structural Analysis System (GSAS) software [71,72].

**Figure 5:**
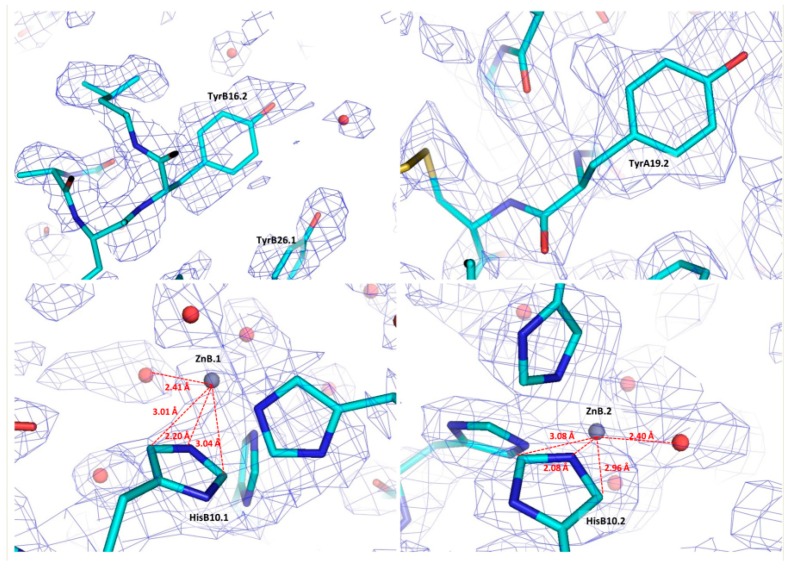
Regions of the final structural model of bovine insulin in stick representation and the corresponding OMIT map contoured at 1σ. The cyan, blue and red colours in the stick representation illustrate C, N and O atoms of different amino acids, while Zn atoms and water molecules are denoted as grey and red spheres, respectively. The closest distances between the two Zn ions and the neighbouring His residues and water molecules are indicated in red dashed lines (Reproduction of Figure 10 from reference [70]. Reproduced with permission of the International Union of Crystallography).

Selected regions of the refined coordinates and the total OMIT map [73] computed at the final steps of analysis are presented in Figure 5. Each of the two zinc ions in the hexameric structure is octahedrally coordinated by three N^ε2^ atoms of three symmetry-related HisB10 residues and three symmetry related water molecules (PDB code: 4IDW).

The successful identification of the above formulations has reinforced the use of powder diffraction, by our group, as a rudimentary tool in daily research, for investigating the structural behavior of HI in a wide range of crystallization conditions in terms of pH and addition of ligands.

### 2.3. Cocrystallization of HI with Phenolic Derivatives and pH Dependence

Phenol and phenol-like compounds have been added in insulin formulations as antibacterial agents since the earliest years of production. It is well known that phenol binds in pockets of the insulin hexamer and alters intensively insulin’s conformation, driving it to the R state [22].

While varying the pH in the presence of phenolic derivatives, a series of phase transitions has been reported. Specifically in the case of cocrystallization with phenol, four distinct polymorphs have been identified, three polymorphs with resorcinol, two with *m*-cresol, and 4-nitrophenol and six with 4-ethylresorcinol (Table 3).

The quality of the obtained data allowed for successful indexing, using the fitted positions of at least 20 first reflections of each diffraction profile. From the extracted data, symmetry and unit-cell parameters were effectively determined.

**Figure 6:**
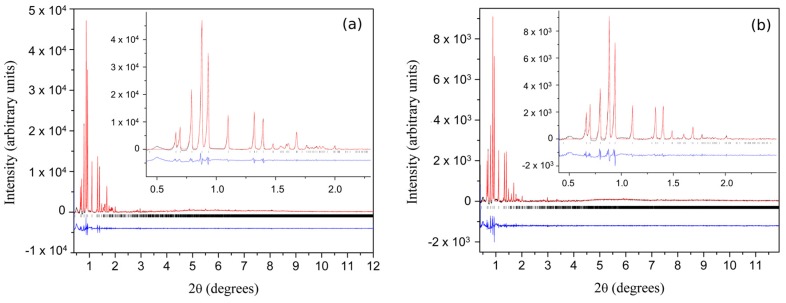
Pawley fit of synchrotron data (European Synchrotron Radiation Facility (ESRF), λ = 1.299825 (16) Å, room temperature (RT)) obtained from Human Insulin (HI) samples belonging to the *P*2_1(α)_ phase, crystallized in the presence of (**a**) phenol, at pH 5.70, or (**b**) resorcinol, at pH 5.29. The black, red and lower blue lines represent the experimental data, the calculated pattern and the difference between them, respectively, while black vertical bars correspond to Bragg reflections compatible with *P*2_1_ space group (Reproduction of Figure 6 from reference [29]. Reproduced with permission of the International Union of Crystallography).

When HI was crystallized with phenol, in addition to the earlier identified polymorphs *C*222_1_, *C*2 [55], and *P*2_1_ [28], a new monoclinic phase of insulin has been detected (Figure 6) within the pH range 5.47–5.70, space group *P*2_1_, (referred thereafter as *P*2_1(__α)_). Indexing of this unit cell was particularly challenging due to dominant-zone problem, as the majority of low two-theta reflections belong to the dominant zone in reciprocal space. These reflections initially were not detected owing to peak overlap, however, combined use of diffraction data collected with different detectors confirmed the existence of a screw axis, and led to the identification of the monoclinic cell *P*2_1(__α)_ with remarkably large unit-cell parameters *a* = 114.682 (6) Å, *b* = 337.63 (2) Å, *c* = 49.270 (4) Å, *β* = 101.555 (6)°, which originally caused the dominant zone effect. Diffraction profiles acquired from *P*2_1(__α)_ crystals extended to ~7.5 Å resolution. This was the first report of this specific crystallographic phase of human insulin.

HI exhibited similar behavior as with phenol, when crystallized with resorcinol at pH 5.29 and 5.46, yielding the same monoclinic phase (space group *P*2_1_, unit-cell parameters *a* = 114.0228 (8) Å, *b* = 335.43 (3) Å, *c* = 49.211 (6) Å, *β* = 101.531 (8)°).

The discovery of a previously unknown crystal form of insulin was the result of a systematic study of the effect of pH—even around its isoelectric point (~5.9)—on the crystallization behavior of insulin in complex with zinc and a phenolic ligand. Nearby the pI region, its solubility is lowest and growing macroscopic crystals suitable for single-crystal X-ray structure determination is least likely to succeed. The novel insulin crystal packing, was identified in this exact pH area in the presence of phenol or resorcinol through XRPD, and that is (probably) the reason why the monoclinic *P*2_1(__α__)_ conformation remained undetected even though crystallization experiments with phenol and resorcinol have been earlier reported [20,27].

Nevertheless, the earlier identified insulin forms (*C*222_1_ and *C*2) were obtained in these studies as well. Human insulin crystallized in the presence of phenol (pH 5.93–6.54), and resorcinol (pH 5.93–7.45) produced crystals with orthorhombic symmetry (space group *C*222_1_) containing three protein hexamers per asymmetric unit [55].

In both cases, the pH increment caused slight lattice parameter alterations, as illustrated by the smooth anisotropic shifts in the peak positions and no indication of a first-order phase transition. Apart from the *C*2 phase, which was only observed during cocrystallization with phenol, all other phases obtained, coincided in crystallization experiments with the two ligands exhibiting minor alterations in unit-cell parameters.

Although phenol and resorcinol can substitute each other as allosteric ligands of the insulin hexamer without detectable changes in insulin structure [28], the presence of ligand apparently influences the crystallization behavior. This is noteworthy, concerning that phenolic binding sites are far from the interfaces or the location of crystal contacts. Results from the systematic screening of crystallization conditions suggest that human insulin crystallized in the presence of phenol and resorcinol is greatly affected by pH. This analytical approach further extends the applicability of powder diffraction methods for efficient macromolecular crystal screening. Specifically, when synchrotron XRPD patterns are employed in the analysis, the low instrumental contribution to the diffraction peaks, resulting in accurate peak positions, allows for high precision in unit-cell parameters determination, and thus small variations of lattices can be quantified precisely.

The structural behavior of HI when cocrystallized with two widely used phenol-based ligands, *m*-cresol and 4-nitrophenol was further examined in a broad pH range [56]. These organic additives, were selected as they can serve as bactericidal agents and earlier structural results on HI complexed with these exist in the literature [27,28,29]. Particularly *m*-cresol comparing to phenol, seems to be a more effective germicide, and is widely used as an antimicrobial preservative in pharmaceutical formulations [74].

**Figure 7:**
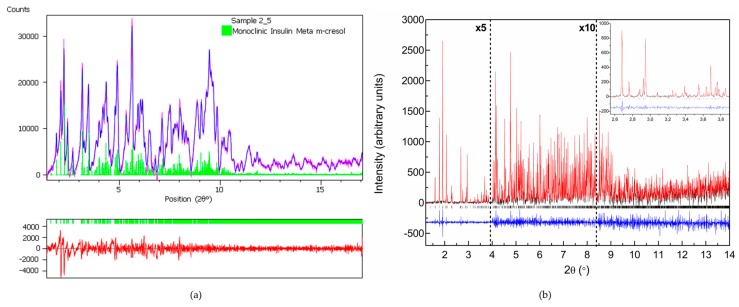
Pawley fit of (**a**) laboratory (λ = 1.541874 Å, RT) and (**b**) synchrotron (ESRF, λ = 1.29994 (1) Å, RT) data of HI crystallized in the presence of m-cresol resulting in monoclinic phase *P*2_1(__γ)_: (**a**) The pink, blue and lower red lines, represent the experimental data, the calculated pattern and the difference between them, respectively. The green bars (lower panel) correspond to Bragg reflections compatible with this monoclinic phase. (**b**) The black, red and lower blue lines represent the experimental data, the calculated pattern and the difference between them, respectively. The vertical black bars correspond to Bragg reflections compatible with this monoclinic form. The profiles have been expanded by a factor of five at Bragg angles larger than 4° and by a factor of ten at angles larger than 8.5° (Reproduction of Figures 5 and 6 from reference [56]. Reproduced with permission of the International Union of Crystallography).

Several polycrystalline samples were produced, and consecutive data collection experiments were performed using various X-ray sources to exploit their influence on diffraction patterns and to ensure the validity of the results. A thorough data analysis revealed a first order phase transition with pH variation, resulting in two distinct polymorphs in both cases (Table 3), whereas a novel monoclinic phase of insulin was identified (space group *P*2_1_, referred in the following as *P*2_1(γ)_). Specifically when HI was crystallized with *m*-cresol (pH range 4.50–6.70) or 4-nitrophenol (pH range 5.1–6.3), this new monoclinic polymorph was identified (Figure 7) with the following lattice parameters, *a* = 87.0749 (7) Å, *b* = 70.1190 (5) Å, *c* = 48.1679 (5) Å, *β* = 106.7442 (8)°. The diffraction patterns obtained for the *P*2_1(__γ)_ polycrystalline samples yield a d-spacing of approximately 6.8 Å.

**Figure 8:**
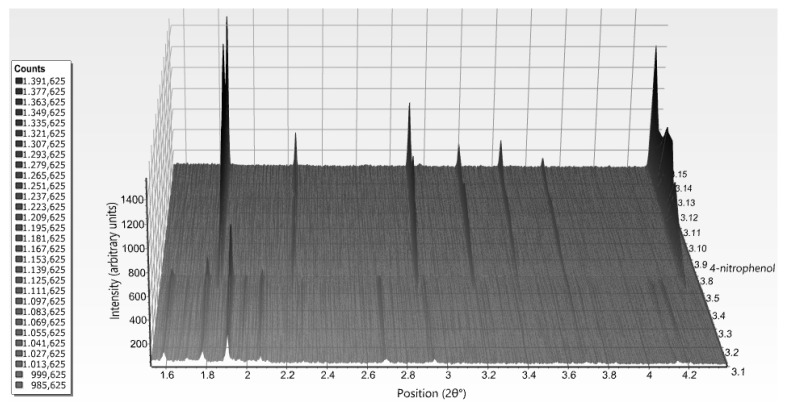
Surface plot of 15 XRPD profiles of HI in the presence of 4-nitrophenol corresponding to the *P*2_1(γ)_ (pH 5.1–6.3) and R3 (pH 6.2–8.1) polymorphs. Data were collected on ID31 (now ID22; λ = 1.29989 (3) Å, RT) (Reproduction of Figure 10, from reference [56]. Reproduced with permission of the International Union of Crystallography).

While moving towards neutral or basic pH regions, a first-order transition occurs, as it is evident in Figure 8. The monoclinic symmetry transforms into a rhombohedral symmetry (space group *R*3) that is stable over a wide pH range (approximately 6.2–8.1) consisting of three protein hexamers per unit cell.

Data analysis of XRPD profiles of HI cocrystallized with 4-nitrophenol, led to the accurate extraction of the following lattice parameters *a* = 80.721 (1) Å, *c* = 37.8039 (5) Å, *γ* = 120.000° for the sample crystallized at pH 6.41. From the parameters obtained it is derived that HI cocrystallized with this ligand acquires the T_3_R_3_^f^ conformation [19]. XRPD profiles collected on ID31 (now ID22) for these samples extended to a resolution of 3.6 Å.

When *m*-cresol is employed in insulin crystallization at pH 6.7–8.6, the *R*3 space group is identified with slightly altered unit-cell parameters. Pawley analysis of high-resolution diffraction profiles resulted in: *a* = 80.0644 (6) Å, *c* = 40.8396 (3) Å, *γ*= 120.000° for the sample crystallized at pH 8.15. These values indicate that HI acquires the R_6_ conformation [28]. XRPD profiles collected on ID31 (now ID22) for these samples extended to a d-spacing resolution of 3.7 Å.

Thorough examination of the lattice parameters close to the transition from the monoclinic crystal type to the rhombohedral one, yields a decrease in the unit-cell volume of about ΔV(*_P_*_2__1(γ)_→*_R_*_3_)/V*_P_*_2__1(γ)_ = −25.53%, while for HI complexed with *m*-cresol the cell is reduced by ΔV(_P2__1(γ)_→*_R_*_3_)/V*_P_*_2__1(__γ__)_ = −21.89%.

Comparing to the isosymmetrical polymorph, *P*2_1(__α)_, identified by Karavassili et al. in 2012 [29], which exhibited remarkably large cell dimensions concerning *a* and *b* axes (*a* = 114.0228 (8) Å, *b* = 335.43 (3) Å, *c* = 49.211 (6) Å, *β* = 101.531 (8)°), the lattice parameters of this new polymorph *P*2_1(__γ)_ are significantly shorter, approaching the already known range of dimensions that other known monoclinic cells adopt [22,28]. Between these two monoclinic forms and the already deposited in the Protein Data Bank *P*2_1(__β)_ (PDB code: 1EV6; [28]), an unusual crystal packing for the *P*2_1(__γ)_ polymorph is noteworthy. While *P*2_1(__β)_, consists of six molecules per asymmetric unit, and 48% solvent content, according to Matthews Coefficient calculation [75,76], the novel *P*2_1(γ)_ polymorph contains twelve molecules per asymmetric unit and 39% solvent content (Matthews coefficient = 2.03 Å^3^·Da^−1^). This difference between the cell contents among the two polymorphs, reveals a denser crystal packing in the case of *P*2_1(__γ)_ which could be of great pharmacological importance.

Interhexamer interactions that may form owing to the very dense packing of the polymorph could associate with enhanced physicochemical properties whereas in the case of crystalline insulin formulations this can be interpreted as increased stability, and thus provide a prolonged formulation lifetime. This could be a key point with a significant impact in the formation of new types of insulin-based microcrystalline preparations for treating diabetes. Furthermore, the preparation of pharmaceutical products consisting of crystals with high protein concentration could lead to minimization of injection times.

The complete structure determination of the novel *P*2_1(__γ)_ polymorph has been derived from the combined use of traditional single-crystal and emerging XRPD approaches and will be presented in a forthcoming publication by our team [77].

The ligand 4-ethylresorcinol, a strong antiseptic and disinfectant of pharmaceutical formulations, was used during systematic crystallization experiments of HI in the presence of zinc ions as well [53]. Diffraction patterns obtained from several sources from crystals grown within the pH range 4.50–8.20 revealed four different crystalline polymorphs (Table 3). Among these, the two new monoclinic symmetry phases (*P*2_1(__α)_ and *P*2_1(γ)_) described earlier, were detected again, emphasizing their characterization as potential targets for the future development of microcrystalline insulin drugs.

**Figure 9:**
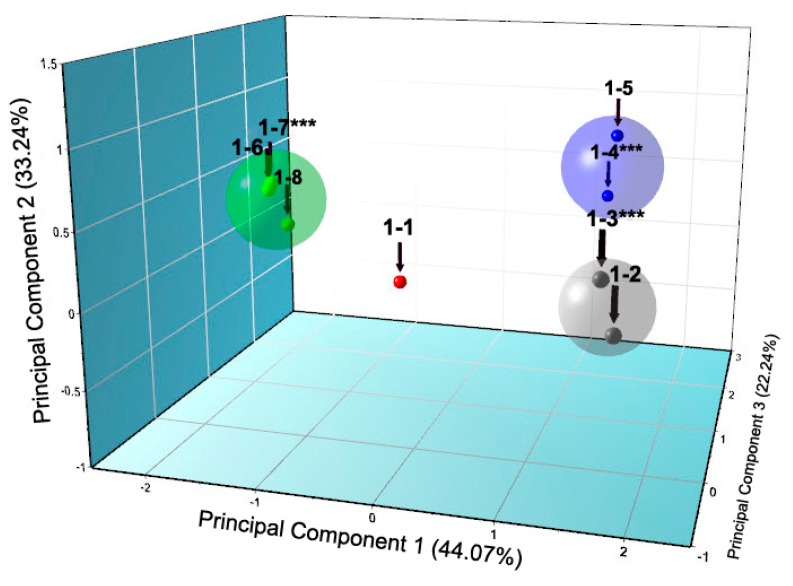
Principal Component Analysis (PCA) analysis of synchrotron (ID31 (now ID22), ESRF, λ = 1.29994 (1) Å, RT) XRPD data of HI crystallized in the presence of 4-ethylresorcinol. Analysis resulted in four discrete clusters (red, grey, blue and green) corresponding to the four different phases identified *P*2_1(γ),_ *P*2_1(α)_, *C*2 and *P*2_1(β)_, respectively. Numbers above each element correspond to sample coding (Reproduction of Figure 4 from reference [57]).

The large quantity of diffraction patterns derived in this study were initially handled via PCA using HighScore Plus software [78], which classified patterns in four distinct groups (Figure 9), corresponding to the mentioned crystalline phases, and indicated also the most representative sample of each cluster (marked with ***).

Systematic data analysis confirmed the three first order phase transitions with pH variation, observed in PCA analysis, which resulted in four distinct polymorphs of monoclinic symmetry (space group *P*2_1_ and *C*2). Accurate unit-cell parameters of each polymorph are presented in Table 3.

**Figure 10:**
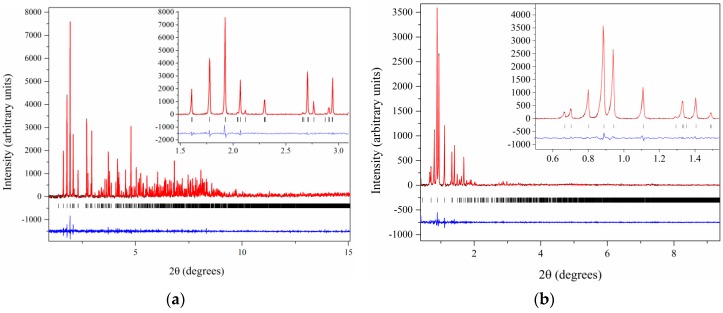
Pawley fit of two diffraction patterns of HI cocrystallized with 4-ethylresorcinol collected on ID31 (now ID22) at ESRF (λ = 1.29994(1) Å, RT) corresponding to: (**a**) Polymorph *P*2_1(γ)_ crystallized at pH 5.02. (**b**) Polymorph *P*2_1(__α)_ crystallized at pH 5.80_._ The black, red and lower blue lines represent the experimental data, the calculated pattern and the difference between the experimental and calculated profiles, respectively. The vertical bars correspond to Bragg reflections compatible with space group *P*2_1_ (Reproduction of Figures 5 and 8 from reference [53]).

Specifically, when HI was crystallized in the presence of 4-ethylresorcinol, within the pH range 4.95–5.80, two novel polymorphs with monoclinic symmetry (*P*2_1(__γ__)_, in pH range 4.95–5.60 with lattice parameters *a* = 87.1323 (8) Å, *b* = 70.294 (2) Å, *c* = 48.064 (2) Å, *β* = 106.1729 (8)° and *P*2_1(__α__)_ in pH range 5.65–5.80 with lattice parameters *a* = 114.130 (7) Å, *b* = 336.086 (3) Å, *c* = 48.987 (5) Å, *β* = 101.935 (8)°) were observed (Figure 10). These crystalline polymorphs had been identified earlier by XRPD [29,56]. Diffraction profiles acquired for the *P*2_1(__γ__)_ polycrystalline samples extended to a resolution of ~6.5 Å, whereas the lower resolution range for the *P*2_1(__α__)_ polycrystalline samples (~112–12 Å) was sufficient for successful indexing and Pawley analysis.

According to Matthews coefficient calculations [75,76], the *P*2_1(__γ__)_ phase contains 12 molecules (two hexamers) per asymmetric unit and doubled molecules per unit cell, corresponding to ~39.3% solvent content (Matthews coefficient of 2.03 Å^3^·Da^−1^). The volume of the cell while shifting from *P*2_1(__γ)_ to *P*2_1(__α)_ increases of about 6.5-fold resulting in a significant unit-cell modification. Summarizing these results in terms of cell volume, the *P*2_1(__α)_ is one of the largest phases that has been identified to date through XRPD, while the *C*222_1_ phase is being sorted as the second one (V_(*C*222__1)_ = 3.054.394 (63) Å^3^ [55], V_[*P*2__1(__α__)]_ = 1836620 (73) Å^3^ [29]). Furthermore, from the crystallization of HI in the presence of 4-ethylresorcinol in the pH range ~6.00–8.00 the crystals obtained, belonged to monoclinic symmetry (space group *C*2 (pH 5.93–6.25) and *P*2_1(__β)_ (pH 6.73–8.05)). The complete structural characterization of these polymorphs has been determined and thoroughly described previously (PDB code 2OLZ [55] and PDB code 1EVR [22,28]).

The systematic crystallization experiments of HI in the presence of 4-ethylresorcinol within the pH range 4.5–8.2 resulted in a discrete characterization of the observed polymorphs in terms of crystal symmetry and lattice parameters. Insulin in these polymorphs adopts the R_6_ molecular conformation of B chain, where binding interactions of ligands in the phenolic pockets seem to stabilize the specific conformation; a process assisted by a number of certain anions such as halides, pseudohalides and organic carboxylates.

This conformation is commonly apparent in pharmaceutical preparations, as most of them contain phenolic derivatives as disinfectants, driving HI either to the T_3_R_3_^f^ or R_6_ molecular conformations [79,80]. Concerning that stability levels increase from T to the R state [79], the existence of the most stable conformations in formulations can serve two principal aspects: sufficient storage stability of the pharmaceutical preparations, and gradual release of the active monomer once the formulation is injected into the human body. Moreover, the allosteric transition at the level of monomer could be proven as essential for the binding affinity of insulin to its receptor [81].

These observations could be of great importance with regard to the improvement of injected preparations, as by reducing crystal’s dissolution rate and increasing the amount of active ingredient per dose would result in more effective formulations. Variations in the pH, during crystallization procedures, can induce the formation of distinct polymorphs with different physicochemical properties such as density, solubility, and stability [82]. These characteristics can further affect the dissolution rate, and thus the bioavailability of the final pharmaceutical products. Therefore, the identification of novel crystalline polymorphs could aid towards optimizing existing formulations, or designing advanced preparations with improved action and characteristics, in accordance with patients’ needs, including preparations associated with alternative methods of administration, such as formulations with sustained release or formulations for inhaled administration [37].

**Figure 11:**
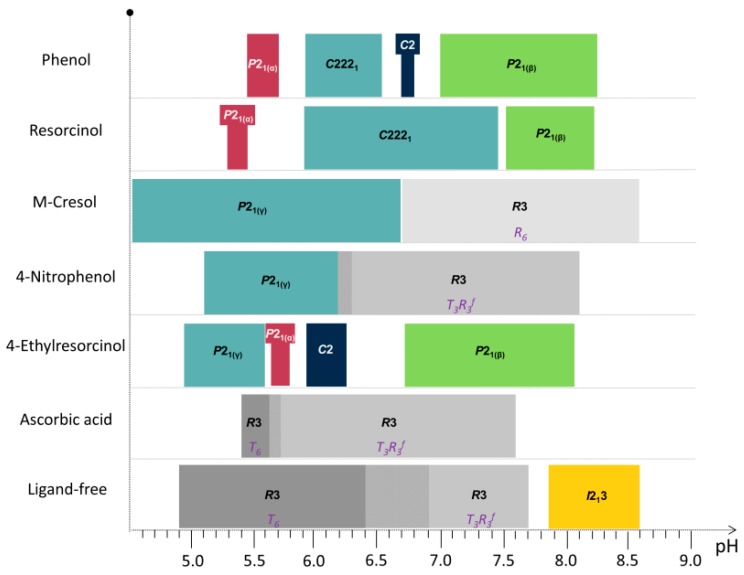
Phase diagram of HI polymorphs obtained in the presence of distinct ligands in the selected pH range provided in Table 3. Shaded regions between distinct crystalline forms denote areas of co-existing polymorphs. Different molecular conformations obtained in the rhombohedral symmetry are shown in italics. Exact pH values within each polymorph occurs are listed in Table 3.

Several HI polymorphs described in this study are summarized in Figure 11, with respect to the ligand and the pH values each polymorph appears.

### 2.4. Cocrystallization of HI with a Non-Phenolic Derivative and pH Dependence

It is well reported both from experimental and clinical studies that oxidative stress plays an essential role in the pathogenesis of diabetes mellitus [83,84], and causes complications affecting the vascular system, kidney, retina, lens, peripheral nerves, and skin [85].

Oxidative stress is characterized by excessive formation or/and the inadequate removal of highly reactive molecules, such as reactive oxygen species (ROS) [86]. Free radicals are excessively produced in diabetics by glucose oxidation, or from other reactions such as nonenzymatic glycation of proteins, and the subsequent oxidative degradation of glycated proteins, all of which further overload the antioxidant system of patients. Thus, there is a necessity for introducing an overall treatment for controlling simultaneously insulin and antioxidants levels to minimize the diabetic’s complications.

One of the strongest antioxidant substances [87], ascorbic acid (vitamin C), was selected for cocrystallization with insulin, replacing the widely used phenol-based ligands (toxic in high concentrations). To date, results indicate that HI has successfully cocrystallized with ascorbic acid in a pH range from 5.4 to 7.6. These new HI-ligand complexes could provide both insulin and free radical scavenger release over a certain period of time after entering blood stream while the beneficial effects of ascorbic acid in diabetes mellitus, and its health complications have been already demonstrated [88,89].

**Figure 12:**
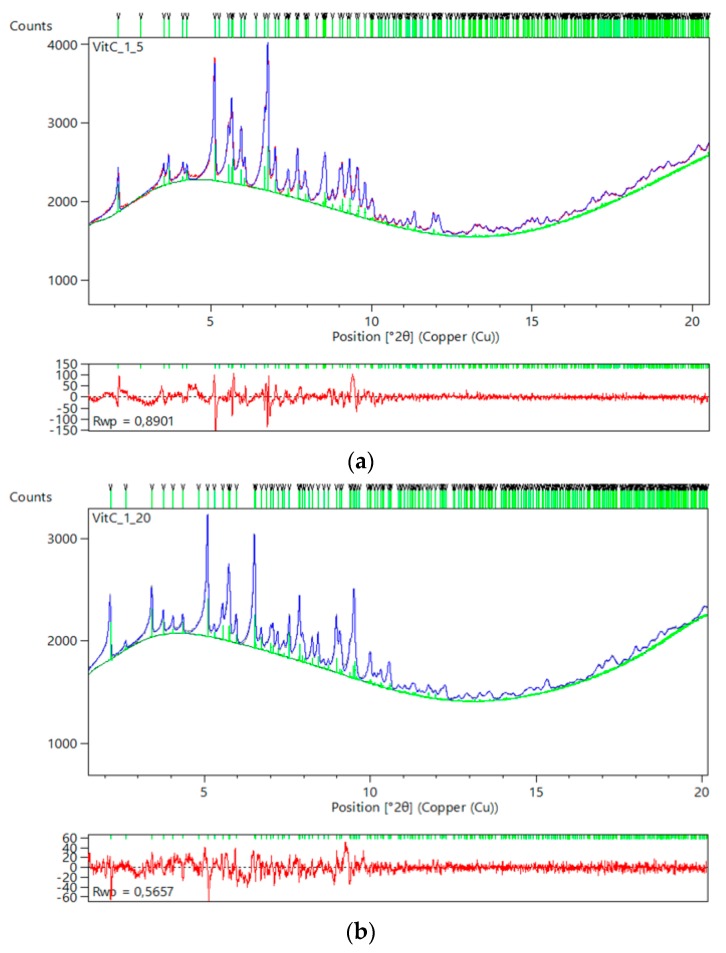
Diffraction patterns of HI cocrystallized with ascorbic acid, in (**a**) T_6_, (**b**) T_3_R_3_^f^ insulin conformation, collected using in-house source (X’Pert PRO, λ = 1.541874 Å, RT). The red, blue and lower red lines represent the experimental data, the calculated pattern and the difference between them, respectively. The green bars (upper and lower panel) correspond to Bragg reflections compatible with this particular space group (*R3*).

Structural characterization of samples, in terms of unit-cell symmetry and dimensions, was performed via XRPD measurements employing both laboratory and synchrotron radiation. In the case where HI was cocrystallized in the pH range 5.40–5.65, diffraction patterns were typically indexed revealing the T_6_ insulin conformation (space group *R*3, *a* = 82.427 (8) Å, *c* = 37.742 (2) Å, for the sample crystallized at pH 5.44). Samples prepared in the pH range 5.70–7.66 were also found to adopt the rhombohedral symmetry (space group *R*3), however, extraction of unit-cell parameters indicates that insulin hexamers comprise the T_3_R_3_^f^ conformation (*a* = 80.686 (6) Å, *c* = 37.5868 (1) Å, for the sample crystallized at pH 7.46). Patterns are shown in Figure 12.

### 2.5. Ligand-Free Crystalline HI Studies and pH Dependence

Towards the direction of understanding better the effect of pH upon HI conformational changes, further crystallization experiments were performed in a wide pH range (4.88–8.56) without the presence of any ligand.

Specifically, HI was crystallized using a solution of 13.14 mg/mL protein concentration, in the presence of 0.8 mM zinc acetate, 10.25 mM sodium thiocyanate, and 0.4 M sodium/monopotassium phosphate buffers of ascending pH per sample, in order to investigate the influence of pH on insulin crystallinity and conformation.

**Figure 13:**
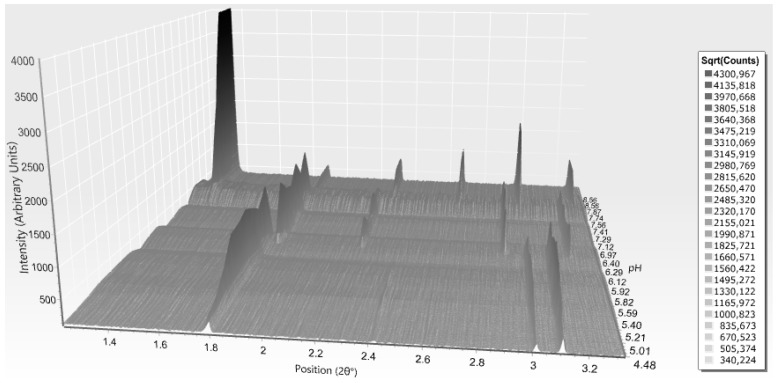
Surface plot illustrating the evolution of diffraction patterns from HI polycrystalline samples in the absence of ligands, at low 2θ range, while varying the pH (5.00–8.60). Patterns were collected at ESRF (ID22, λ = 1.29974(6) Å, 280 K) [90].

Diffraction patterns from both synchrotron and laboratory sources were collected and indexed successfully. Systematic data analysis led to the identification of 2 different space groups; *R*3 rhombohedral (pH range 4.9–7.7), and *I*2_1_3 cubic (pH range 7.75–8.60). Within the acidic pH, the T_6_ configuration (space group *R*3, *a* = 82.9943 (5) Å, *c* = 34.0642 (2) Å) was identified while in pH range 6.9 to 7.7, the T_6_ alters to T_3_R_3_^f^ (*a* = 80.6630 (5) Å, *c* = 37.7459 (2) Å), a transition that is evidently depicted in peak’s positions changes (Figure 13). The figure indicates an additional structural modification from samples with even higher pH values (7.8–8.6). A first order phase transition occurs at pH around 7.7, and insulin molecules obtain a cubic symmetry (space group *I*2_1_3, *a* = 78.9 Å, PDB code: 9INS) [30].

**Figure 14:**
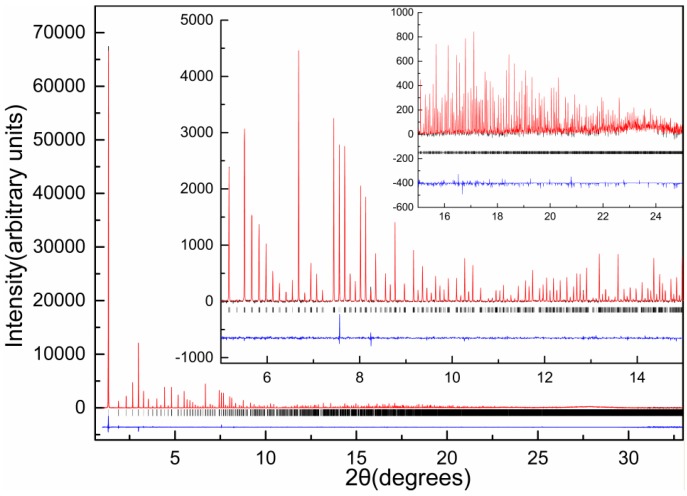
Pawley fit of diffraction pattern of HI crystallized at pH 8.56 (space group *I*2_1_3), collected on ID22 at ESRF (λ= 1.29974(6) Å, 280 K). The black, red and blue lines represent the experimental data, the calculated pattern and the difference between the experimental and calculated profiles, respectively. The vertical bars correspond to Bragg reflections compatible with space group *I*2_1_3. Insets correspond to magnifications of the fit in selected 2θ ranges [91].

All diffraction patterns of this study were collected on ID22 at the ESRF, and extend to a resolution of 3.3 Å (*R*3 polymorphs) and 2.7 Å (*I*2_1_3 polymorph) as illustrated in Figure 14.

**Figure 15:**
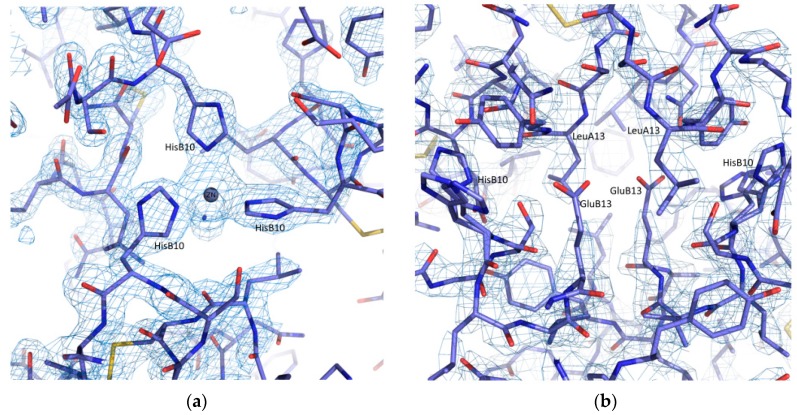
Selected regions of refined structural models and OMIT maps contoured at 1σ showing (**a**) The arrangement of three HisB10 residuesaround the Zinc ion in rhombohedral symmetry (space group *R*3, pH 7.29). (**b**) The arrangement of HisB10 residues in cubic symmetry (space group *I*2_1_3, pH 8.56), where the absence of zinc ions is evident. In the cubic phase, HisB10 is flexible occupying 3 conformations as shown in the right panel [91].

HI crystals grown in solutions with pH higher than 7.7 adopt the cubic symmetry, which is the most common zinc-free crystal form, in accordance with bibliography [92,93]. However, zinc ions, mandatory for HI hexamer formation [94], were initially added during crystallization. Consequently, we conclude that in alkaline conditions, zinc ions are not able to interact with the molecule, and for this reason HI crystals are formed from dimers and not from hexamers. The accuracy of this allegation was verified via a structure solution of a microcrystalline sample at pH 8.56 from powder diffraction data (d-spacing resolution ~2.5 Å), which clearly revealed the absence of zinc ions from their common binding sites (Figure 15): two identical high-affinity sites located on the three-fold symmetry axis near histidines in the two distinct symmetries. Detailed description of the cubic structure will be discussed elsewhere [91].

This phenomenon can be explained by considering the charge of all different ionizable groups of insulin molecule. For pH ≥ 7.5, histidines, due to imidazole rings’ acid dissociation constant pKa (7.5), are neutral [95]. Uncharged His cannot associate with zinc ions, and consequently insulin hexamers cannot form.

This observation could be of great importance for the pharmaceutical industry. The majority of the commercially available compounds consist of crystals containing HI hexamers, tightly packed within the unit-cell, allowing a minimum amount of solvent. However, it is evident that even slight alterations in storage conditions (e.g., temperature), which can directly affect parameters such as pH, may alter the tertiary molecular structure modifying physicochemical characteristics of the molecule and drug’s ADME.

## 3. Discussion

The present review reports recent research advances of insulin-based polycrystalline compounds as potential therapeutics against diabetes. The majority of structural studies reported were conducted by employing macromolecular powder diffraction, a powerful complementary tool for swift and accurate structure determination of powder crystalline material.

Continuous improvements in protein engineering, as well as the development of insulin analogs, introduced in the market an important number of compounds that are capable of mimicking, up to a certain level, the physiological secretion of insulin, accommodating both basal and prandial necessities. Empowered by the constant increase of diabetes cases among the population and the degenerative nature of the disease, the insulin market has grown at a 7% annual rate during the past decade [96]. This fact further motivates the worldwide drive for producing new insulin formulations and delivery systems in addition to the large portfolio of insulin products and analogs currently available from several manufacturers.

However, new products need to be studied structurally to reveal specific characteristics and polymorphism [97], defining physiological properties and clinical efficiency of formulations. This enormous need for structural data has consequently led to a parallel development of techniques and approaches such as macromolecular powder diffraction for dynamic extraction of information even under challenging circumstances. In the last eighteen years, major advances have been made in the field of XRPD in terms of experimental methods and computational tools strengthening this technique, and significantly expanding the variety of substances and samples that can be examined [45,46,47,52]. XRPD data collection is simple, providing a distinct diffraction pattern for each polymorph within few minutes. In addition, polycrystalline material can be studied in unity, rather than by isolating individual crystals to perform experiments, and thus XRPD methods can offer an integrated exploration of the system under investigation, including homogeneity and purity control. Those are exactly the reasons why the technique is pointed out as the most suitable tool for quickly and accurately characterizing numerous microcrystalline suspensions. Furthermore, this review manifests the applicability of XRPD for studies of microcrystalline proteins, and is an ideal technique to be combined with crystallographic studies at X-ray free electron lasers, as well as with electron diffraction methods [98,99,100]. Synergistic use of these techniques will considerably empower past approaches in structural characterization of biological macromolecules, employing micro-sized crystals.

To date, research findings on human insulin microcrystals exhibit a fascinating polymorphism, occurring upon physicochemical modifications of their environment (i.e., pH, ligand binding), and further expanding the already rich phase diagram of the molecule. Four new biologically active types of HI crystals have been identified, and their structures have been successfully determined by a combination of powder and single crystal diffraction measurements. Additionally, studies were performed including cocrystallization of HI with a molecule of already proven pharmaceutical action, towards identifying the most beneficial complexes that will occur, which will lead to microcrystalline products of enhanced stability and activity.

Until the diabetes cure puzzle is completed, research for pharmaceutical products containing microcrystals with improved activity and stability will be at the center of scientific interest worldwide. Leading to a minimization of injection times, these compounds will be a life-quality improvement of great importance for millions of patients.

## Figures and Tables

**Table 1:** Crystallization conditions for the samples used in the study.

	A	B	C	D	E	F	G	H	I	J	K	Χ
Space group	*P*2_1_	*C*222_1_	*I*2_1_3	*R*3	*R*3	*R*3	*R*3	*R*3	*P*4_3_2_1_2	*P*4_3_2_1_2	*P*4_3_2_1_2	*C2*
Insulin (mg·mL^−^^1^)	5.2	3.5	10	14	3.8	3.8	3.8	1.5	3.8	3.8	1.5	3.5
Zn/hexamer	2.3	2.3		2.5	4	2.2	2.2	2.2	3	3	3	2.3
Phenol derivative (mM) ^†^	20 ^1^	25 ^1^		19 ^3^/19 ^4^		65 ^2^	65 ^2^	65 ^2^	7 ^3^/14 ^4^	7 ^3^/14 ^3^	7 ^3^/14 ^3^	25 ^1^
NaCl (M)		1.0	1.0	0.02	0.3	0.12	0.12	0.12				1.0
Na acetate (M)					0.01	0.01	0.01	0.01				
Na citrate (M)				0.11								
Na_2_HPO_4_ (M)	0.48	0.05	0.04						0.013	0.013	0.013	0.05
Urea (M)		1.1										1.1
Tris (M)				0.14								
Protamine									Added in isophane ratio ^‡^			
pH	7.3	6.7	7.2	8.15	5.5	7.4	7.4	7.4	7.3	7.3	7.3	7.0

^†^ Phenol derivatives: ^1^ resorcinol (benzene-1,3-diol); ^2^ methyl *p*-hydroxybenzoate (methyl 4-hydroxybenzoate); ^3^ phenol; ^4^ *m*-cresol (3-methylphenol). ^‡^ Protamine added in isophane ratio: the protamine/insulin molar ratio that results in minimum protein concentration in the supernatant [64,65] (Reproduction of Table 2 from reference [54]).

**Table 2:** Crystallographic properties of all insulin samples employed for structural investigation.

							Unit Cell				
Crystal	λ (Å) ^†^/Beamline	Trade Name	Crystal System	Space Group	Sequence Origin ^‡^	B-Chain Configuration	*a* (Å)	*b* (Å)	*c* (Å)	*β* (°)	PDB Ref.^§^
A	0.97/911-3		Monoclinic	*P*2_1_	H	R_6_	61.3	61.7	47.5	111.3	1EV6 [28]
B	1.00/911-2		Orthorhombic.	*C*222_1_	H	R_6_	58.9	219.4	223.7		In-house database
C	1.00/911-2		Cubic	*I*2_1_3	H	T	78.9	78.9	78.9		1APH [28]
D	0.97/911-3	Detemir	Rhombohedral	*R*3	H	R_6_	78.9	78.9	39.5		1EV3 [28]
E	0.97/911-3		Rhombohedral	*R*3	H	T_3_R_3_^f^	80.6	80.6	37.8		1TRZ [19]
F	0.969/711	Ultralente	Rhombohedral	*R*3	H	T_6_	81.3	81.3	33.7		1MSO [65]
G	0.969/711	Ultratard	Rhombohedral	*R3*	H	T_6_	82.5	82.5	34.0		4INS [18]
H	0.969/711	Lente			B, P						
I	0.969/711	Penmix30	Tetragonal	*P*4_3_2_1_2	H	R_6_	62.9	62.9	85.9		In-house database
J	1.00/911-2	Novomix30	Tetragonal	*P*4_3_2_1_2	H B28Asp	R_6_	62.8	62.8	86.9		In-house database
K	0.969/711	Protaphan	Tetragonal	*P*4_3_2_1_2	P	R_6_	62.9	62.9	85.9		7INS [61]
X	0.97/911-2		Monoclinic	*C*2	H	Unknown	100	60	62	116	

^†^ Wavelength used during data collection. ^‡^ H: human, B: bovine, P: porcine. PDB: Protein Data Bank (www.rcsb.org). ^§^ Coordinate files used for simulated powder patterns (Reproduction of Table 1 from reference [54]).

**Table 3:** Crystallographic properties of human insulin complexes with specific phenol derivatives as extracted from XRPD data (cell values reported, derive from an indicative sample within each pH range).

Phenol Derivative	pH Range	Space Group	Indicative Sample’s pH	*a* (Å)	*b* (Å)	*c* (Å)	*β* (°)	Resolution Range (Å)
phenol								
	5.47–5.70	*P*2_1(__α__)_	5.70	114.682 (6)	337.63 (2)	49.270 (4)	101.555 (6)	112.2–7.5
	5.93–6.54	*C*222_1_	6.14	60.287 (1)	221.797 (6)	228.812 (5)	90	115–7.5
	6.70–6.75	*C*2	6.75	103.0115 (5)	61.3213 (2)	63.5783 (4)	117.2244 (5)	45.9–5.3
	7.01–8.25	*P*2_1(β)_	7.46	61.0920 (4)	61.8279 (4)	47.9302 (4)	110.6253 (7)	45–4.4
resorcinol								
	5.29–5.46	*P*2_1(__α__)_	5.29	114.0228 (8)	335.430 (3)	49.211 (6)	101.531 (8)	112.2–7.5
	5.93–7.45	*C*2221	6.40	60.5579 (7)	220.907 (3)	228.320 (3)	90	115–7.5
	7.53–8.22	*P*2_1(__β__)_	8.22	61.0008 (4)	62.0040 (3)	47.8823 (3)	110.0465 (5)	45–4.4
*m*-cresol								
	4.50–6.70	*P*2_1(__γ__)_		87.0749 (7)	70.1190 (5)	48.1679 (5)	106.7442 (8)	46.5–6.8
	6.70–8.60	*R*3 (R_6_)	8.15	80.0644 (6)	80.0644 (6)	40.8396 (3)	90	40.5–3.7
4-nitrophenol								
	5.1–6.3	*P*2_1(__γ__)_	5.97	87.118 (1)	70.9493 (9)	48.4967 (9)	106.653 (1)	46.5–6.8
	6.2–8.1	*R*3 (T_3_R_3_^f^)	6.41	80.721 (1)	80.721 (1)	37.8039 (5)	90	40.5–3.6
4-ethylresorcinol								
	4.95–5.60	*P*2_1(__γ__)_	5.14	87.132 (3)	70.294 (2)	48.064 (2)	106.259 (3)	47–6.5
	5.65–5.80	*P*2_1(__α__)_	5.80	114.130 (7)	336.086 (3)	48.987 (5)	101.935 (8)	112–12
	5.93–6.25	*C*2	5.97	103.0848 (4)	61.6636 (2)	63.5006 (4)	117.417 (5)	46–7
	6.73–8.05	*P*2_1(__β__)_	6.73	62.8231 (7)	62.1078 (5)	47.8362 (6)	111.6913 (9)	45–6
